# DNA display of glycoconjugates to emulate oligomeric interactions of glycans

**DOI:** 10.3762/bjoc.11.81

**Published:** 2015-05-11

**Authors:** Alexandre Novoa, Nicolas Winssinger

**Affiliations:** 1 Department of Organic Chemistry, NCCR Chemical Biology, University of Geneva 30, quai Ernest Ansermet, 1211 Geneva, Switzerland

**Keywords:** glycan, glycoconjugate, DNA display, multivalency, nucleic acid conjugates, oligomeric interaction

## Abstract

Glycans (carbohydrate portion of glycoproteins and glycolipids) frequently exert their function through oligomeric interactions involving multiple carbohydrate units. In efforts to recapitulate the diverse spatial arrangements of the carbohydrate units, assemblies based on hybridization of nucleic acid conjugates have been used to display simplified ligands with tailored interligand distances and valences. The programmability of the assemblies lends itself to a combinatorial display of multiple ligands. Recent efforts in the synthesis and applications of such conjugates are discussed.

## Introduction

**Figure 1:**
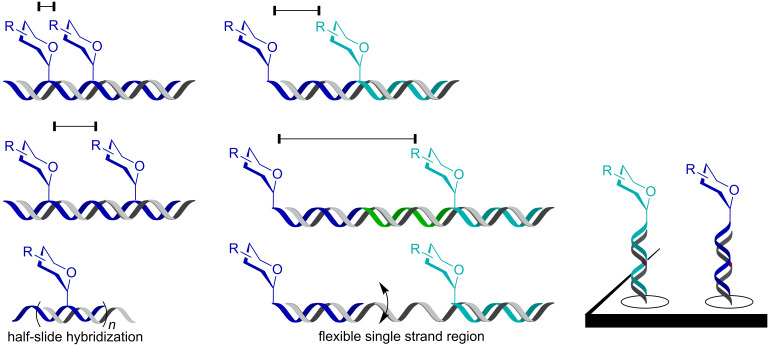
DNA display of glycans.

Cell surface glycans are important actors in cellular recognition and have been implicated in numerous events such as fertilization, embryonic development, lymphocyte trafficking and cancer metastasis [1–4]. In contrast to many small molecule ligands where a functional output is often the product of a single high-affinity interaction with a target macromolecule, glycans’ interactions with glycan-binding proteins (GPB) or lectins are typically low affinity. However high avidity and specificity is achieved through the concerted interactions of multiple ligands with well-defined spatial geometry [5]. The oligomeric nature of these interactions not only provides a mechanism to enhance avidity and specificity but can also trigger a functional output through formation of receptor clusters and membrane deformation [6]. Pathogens frequently use cell surface glycans to gain entry into cells [7]. Accordingly, there is a longstanding interest in tools to manipulate these interactions for structure–function studies and as potential therapeutics [8–9]. The demonstration that the high avidity of oligomeric interactions with cell surface carbohydrates can be outcompeted with a synthetic scaffold that recapitulates the geometry of the oligomeric interactions provided an important precedent [10] and has stimulated intense research in glycomimetics, glycodendrimers, and glycopolymers [11–12]. Over the past decade, there has been a growing interest in using oligonucleotide hybridization [13–15] to scaffold the assembly of glycans in order to tailor spatial geometry [16]. Attractive features of this hybridization-based supramolecular scaffold are that double strand nucleic acid is fairly rigid with well-defined nucleotide spacing and that the valence and ligand combination can be adjusted through the hybridization instructions (Figure 1). This hybridization can be used to rigidify a nucleic acid strand containing multiple glycans at variable positions, to generate oligomers through half-slide hybridization and to combinatorial pair multiple ligands. The flexibility of the template can be further tuned with single strand stretches that remain flexible. Such assemblies can also be generated in spatially addressable format using DNA microarrays. At the core of this technology is the ability to conjugate biologically relevant glycans or glycomimetics to nucleic acids. Herein, we present an update of the different chemistries used in the glycoconjugations and the different strategies used to display the glycans with DNA templates.

## Review

### Glycan–DNA conjugates

**Scheme 1:**
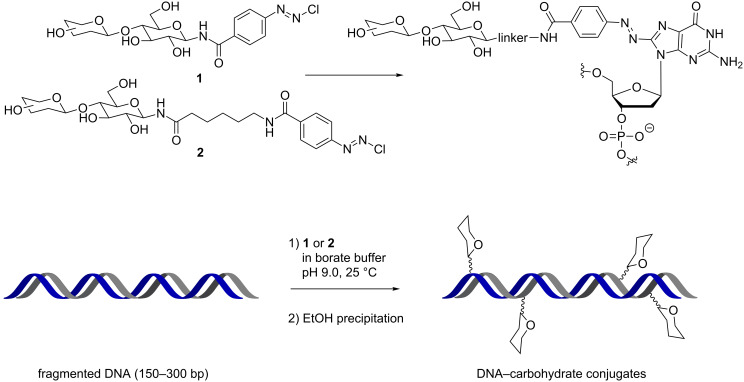
Synthesis of glycoconjugate DNA by diazo-coupling.

An initial solution used in the pioneering work of Kobayashi [17] for nucleic acid–glycan conjugation was the chemoselective reaction of *p-*diazobenzoyl conjugates **1** and **2** with a guanidine nucleotide (G, derivatization at the 8-position) within DNA (Scheme 1) [18]. The conjugation was performed in solution on dsDNA and was used to introduce either lactose or cellobiose moieties (with and without linker). The substitution degree was proportional to the G content of different DNAs and the B-type conformation remained up to a high level of conjugation (25%). Interestingly, the double strand glycosylated DNA showed a higher melting temperature and a stronger enzyme resistance compared to the native DNAs. Most importantly, a superior affinity for the glycan–DNA conjugate (*K*_A_ = 10^4^–10^5^ M^−1^) compared to glycan alone was observed with RCA_120_ lectin (*Ricinus communis* agglutinin) demonstrating the synergy of interactions amongst the glycan units along the DNA.

**Scheme 2:**
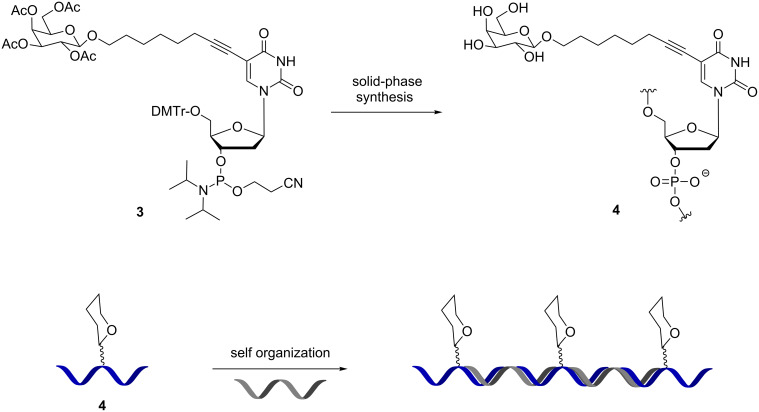
β-Galactose-modified deoxyuridine phosphoramidite used for solid-phase DNA synthesis and DNA display of glycan.

As an alternative strategy to gain better control on the composition of DNA–glycan conjugate, the same group reported the synthesis of phosphoramidite derivatized with an acetyl-protected monosaccharide **3** and its incorporation into DNA to access well-defined DNA conjugates **4** (Scheme 2). Cleavage of the acetate groups occurs upon ammonia treatment for DNA cleavage/deprotection [19–21]. Three galactosylated DNA conjugates with different lengths were obtained and mixed with the corresponding half-slide complementary DNA to obtain supramolecular oligomers forming galactoside clusters. The different assemblies were tested for their binding affinity to the RCA_120_ lectin showing a correlation between affinity and the inter-galactose distance thus establishing that DNA display of glycans can be used to tune the optimal spatial arrangement of the ligands in synergic interactions.

**Scheme 3:**
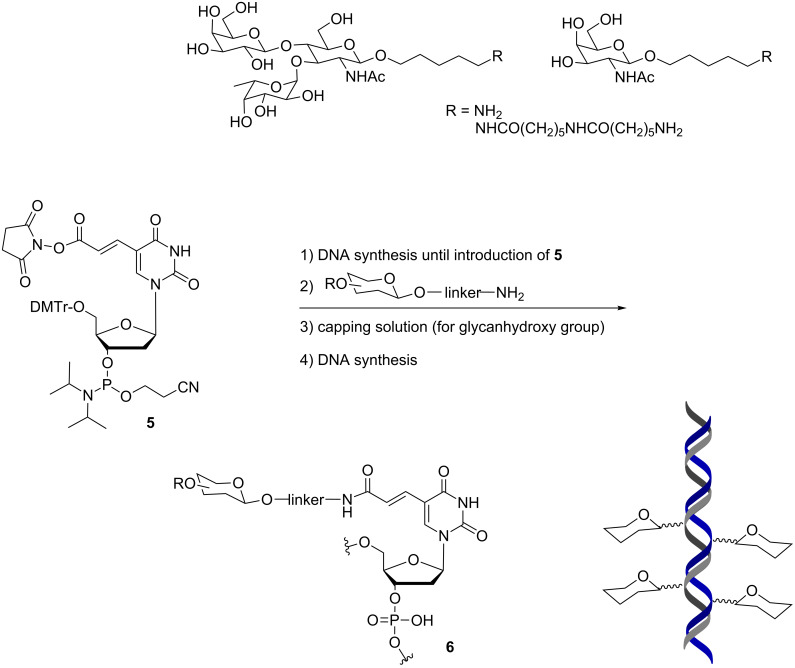
(NHS)-carboxy-dT phosphoramidite as a general entry for the solid-phase synthesis of glycan–DNA conjugates.

This synthetic strategy was further refined by Seeberger and co-workers [22] with the use of commercially available *N*-hydroxysuccinimide (NHS)-carboxy-dT phosphoramidite **5** (Scheme 3). This method allows the sequential introduction of any amine-functionalized glycan during DNA synthesis and was shown to be compatible with more complex glycans such as Lewis X trisaccharide. The capping step in DNA synthesis resulted in acetylation of the glycan thus blocking the glycan’s hydroxy groups. The generality of this method was illustrated with the synthesis of 16 different DNA conjugates containing one or two glycan units. Analysis of glycan-modified duplexes by CD spectroscopy indicated minimal perturbation of the helical structure and thermal stability. Surface plasmon resonance (SPR) affinity measurements with murine C-type lectin receptor (mMGL) showed specific binding only for duplexes containing two or four Lewis-X units.

**Figure 2:**
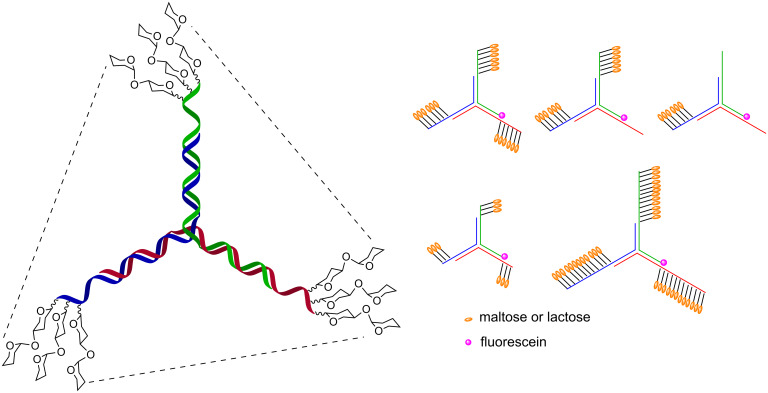
Multivalent triangular glycoDNA assemblies.

Alternatively, Ebara and co-workers have shown that glycan–DNA conjugates can be accessed enzymatically using glycan-functionalized desoxyuridine triphosphate as substrate with KOD Dash DNA polymerase [23]. The applicability of the method was illustrated with the incorporation of multiple units of lactose or maltose in different DNA sequences. The same group used this technology to prepare triangular architectures of glycosylated DNA based on a 3-way junction (Figure 2) [24]. The triangular assemblies were built using 1, 2 or 3 glycosylated DNAs, each with 3, 6 or 12 glycan units. As a proof of principle, the assemblies were tested for their affinity to concanavalin A (ConA). This lectin has 4 binding sites for glucosides and mannosides (preferred) spaced by 72 Å. Titration studies showed a clear dependence on the functionalization of each arm in the 3-way junction consistent with a synergistic interaction of each arm with a binding site. However, the number of glycan units (on each arm 3, 6 or 12) had marginal impact on the binding suggesting a saturation of binding site occupancy. For the structure with 6 units of maltose on each arm, a *K*_D_ of 1 μM was measured which is 700-fold more potent (40-fold per sugar) than monovalent maltose.

The advent of the copper-catalyzed azide–alkyne cycloaddition (CuAAC) [25–26] has naturally inspired the use of this powerful conjugation method to prepare glycan–DNA conjugates.

**Scheme 4:**
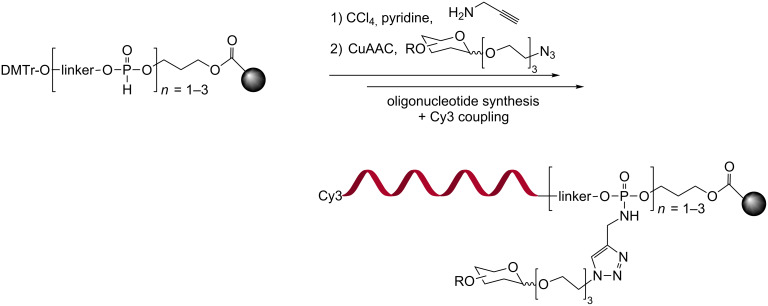
Preparation of the DNA glycoconjugate by CuAAC.

Chevolot and co-workers used this method to conjugate glycans at the 3’-end of DNA [27]. The DNA synthesis was initiated with H-phosphonate that was converted to a phosphoramidate alkyne by oxidative amidation using carbon tetrachloride with propargylamine. The microwave-assisted click-conjugation was performed on a solid phase upon completion of the DNA synthesis (Scheme 4).

**Scheme 5:**
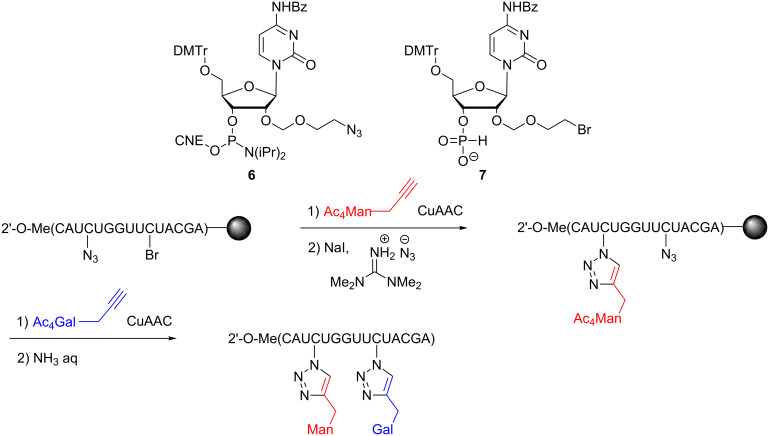
DNA glycoconjugation by sequential CuAAC.

Lönnberg and co-workers prepared a thymidine modified at the 4’-position with an azidomethyl group to achieve conjugation during solid-phase DNA synthesis or in solution post DNA cleavage [28]. Subsequently, the same group reported a method to introduce two different glycans sequentially on the DNA strand at the 2’-position using an azido and a bromo-modified thymidine **6** and **7** (Scheme 5) [29].

**Scheme 6:**
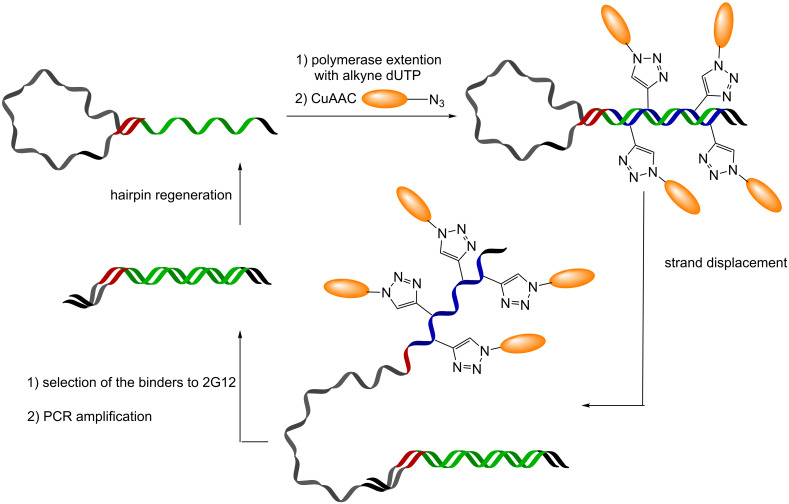
Selection with modified glycoconjugate aptamers (SELMA).

Krauss and co-workers elegantly extended the utility of SELEX [30] to generate aptamers functionalized with glycans through CuAAC [31–32]. Their approach, termed SELMA (selection with modified aptamers), is a multistep procedure that allows screening, selection and amplification of DNA glycoconjugates (Scheme 6). At first, a library of single strand DNA with a hairpin is extended with a polymerase replacing dTTP by an alkyne-modified desoxyuridine triphosphate to give a full hairpin with randomized alkyne groups on one strand. Then, CuAAC is performed with a glycosyl azide and the hairpin is released by strand displacement thus allowing the glycosylated strand to adopt a folded structure. Affinity selection and reiteration of the cycle enables the in vitro evolution of glycan-functionalized aptamers. This technology was used to screen ligands for 2G12, an antibody that neutralizes HIV by binding to the high mannose epitope of gp120. For this purpose, the aptamer library was functionalized with oligomannoses (Man_4_-azide or Man_9_-azide) leading to the selection of glycan-functionalized aptamers bearing 7–14 glycan units and with a *K*_D_ below 220 nM. A mutagenesis study showed that the affinity was also sequence dependent and not uniquely due to the high glycosylation of the DNAs. The tertiary structure of the glycan conjugates predisposed the ligands productively thus resulting in a high affinity. A variation of this strategy using mRNA also yielded peptidoglycans with high affinity to 2G12 [33].

### DNA–PNA glycosylated hybrids

**Scheme 7:**
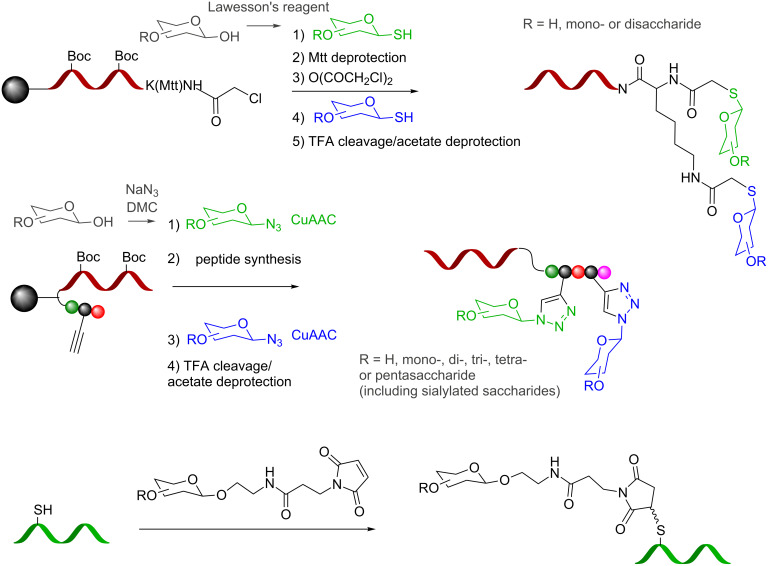
Synthesis of PNA glycoconjugates (Mtt: 4-methyltrityl; R = H or (oligo)saccharide).

As an alternative to DNA, peptide nucleic acid (PNA) [34] has also been used to tag glycans and to program their assembly based on the rules of hybridization. From an assembly standpoint, stable PNA–DNA duplexes can be achieved with shorter sequences than the corresponding DNA homoduplex (10–14mer PNA typically provides sufficient duplex stability) [35]. From a chemistry standpoint, the fact that PNA synthesis involves peptide coupling reactions with a broad arsenal of protecting group combinations facilitates the introduction of functionalities for the conjugation of glycans [36]. The first method reported was leveraged on a nucleophilic coupling between readily available glycosyl thiol (obtained in one step by treatment of a native carbohydrate with Lawesson’s reagent [37]) and a chloroacetamide-functionalized PNA (Scheme 7) [38–39]. Using this method, we have shown that diverse glycans could be iteratively introduced on amino acid linkers. Inspired by Shoda’s activation [40] which provides facile access to complex glycosyl azides from native carbohydrates, we subsequently applied reiterative CuAAC conjugation of glycans on propargyl glycine residues within a peptide [41–42]. Since these methods are compatible with the powerful scheme of mix and split combinatorial chemistry the synthesis of libraries is easily performed wherein each library member is tagged with a unique sequence. Conjugation of glycans at different positions within a PNA oligomer has been achieved by Seitz and co-workers using thiols imbedded in the backbone of the PNA that were chemoselectively conjugated to a maleimide–glycan adduct [43].

**Figure 3:**
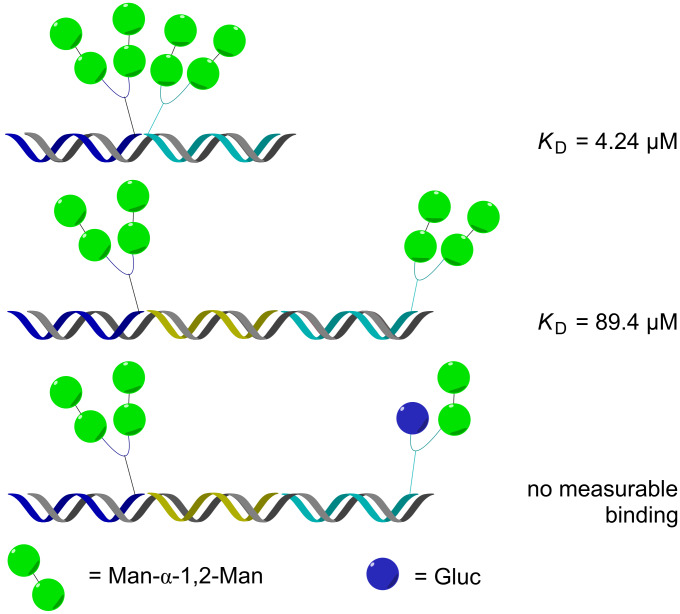
DNA display of PNA-tagged glycans designed to emulate HIV's gp120 epitope.

Our first efforts in the area of glycan display aimed to demonstrate that a DNA template could be used to program the assembly of discrete PNA-tagged ligands in order to recapitulate the geometry of HIV’s gp120 glycan epitope which is composed of multiple copies of a high mannose undecasaccharide [38]. An advantage of displaying ligands through template assembly of discrete units is that the pairing and distance can be controlled though the template instructions. To this end a pilot library of fourteen PNA-tagged glycoconjugates that included mannose disaccharides joined by linkers of different lengths were paired through hybridization, varying the ligand combinations and interligand distances. Measuring the affinity of 32 different assemblies against 2G12, an antibody that neutralize HIV through tight binding with the glycan epitope, showed a clear distance–affinity relationship that was consistent with the proposed antibody–epitope interaction. Notably, neither of the PNA-tagged fragments making up the highest affinity assembly had measurable affinity for the antibody thus demonstrating a clear synergy in the interaction of the templated fragments (see Figure 3 for selected examples).

**Figure 4:**
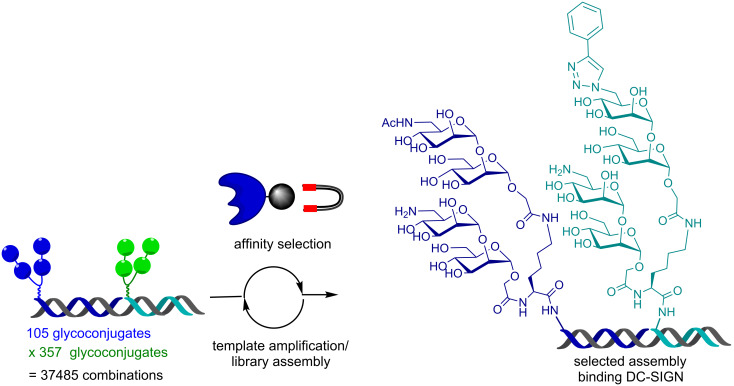
Combinatorial assembly and selection of two PNA glycoconjugate libraries on DNA templates.

This approach was subsequently scaled out to optimize the affinity of DC-SIGN ligands using a library of PNA-tagged glycans that included unnatural modifications in the glycans. DC-SIGN is a tetrameric lectin implicated in interactions with a broad array of pathogens including HIV. A library of 37,485 assemblies was prepared by hybridization of two sets of PNA-tagged glycoconjugates onto a library of DNA templates (Figure 4). Screening the library by affinity selection against immobilized DC-SIGN and analysis of the best-fit sample by PCR amplification/sequence analysis of the template led to the discovery of an assembly with a 30-fold enhancement in binding over the unmodified mannose assembly [44]. Importantly, following PCR amplification of the template, the library can be reassembled making the technology compatible with reiterative cycles of selection/amplification.

**Figure 5:**
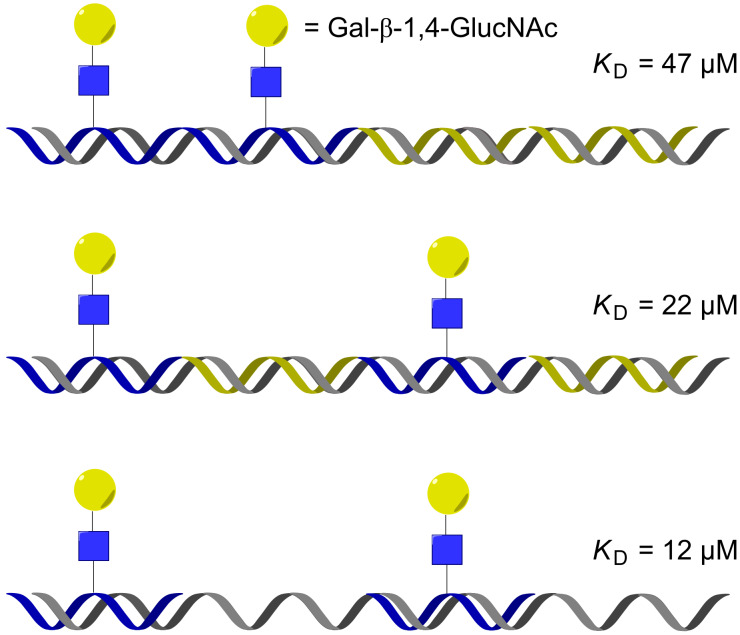
DNA display of ligand bridging opposing binding sites in a lectin (ECL).

Seitz and co-workers demonstrated that a DNA display could be used to interrogate topologically more challenging geometries, namely, bridging binding sites on opposing faces of a lectin. Using a set of five different glycan–PNA conjugates and different DNA templates, the optimal spatial arrangement of the ligands was systematically investigated. Additionally, the flexibility of the PNA–DNA duplexes was also modulated by introducing nick-sites and partially unpaired regions in the DNA display (see Figure 5 for selected examples) [43]. Each assembly was tested for its affinity to ECL (*Erythrina cristagalli* lectin) and the results confirmed that the affinity with the lectin is dependent on the distance between the glycan units (as suggested by crystallography) and benefited from the added flexibility of the linker introduced by an unpaired region. The same approach was also used to identify the optimal spatial arrangement for assemblies targeting RCA_120_ and L-selectin with mannose, LacNAc and sialyl Lewis X–PNA conjugates [45]. The highest binding affinity to RCA_120_ was obtained with a bivalent glycan assembly (LacNAc, presented at 140 Å distance) that was 70 times better than the monovalent assembly. Notably, the enhanced binding affinity for the divalent display is consistent with a distance of ca. 130 Å between the binding sites. It was also demonstrated that DNA-templated displays could be harnessed to combine a DNA-based aptamer with a glycan.

### Glycan arrays prepared by hybridization to a DNA microarray

**Figure 6:**
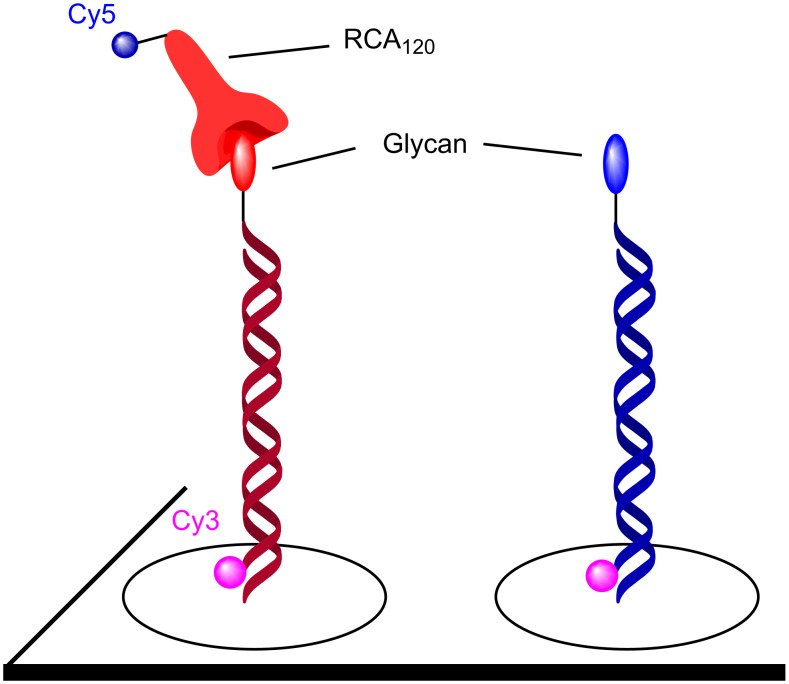
A glycan array prepared by hybridization of glycan–DNA conjugates and screening of RCA_120_.

Microarray technologies have enjoyed tremendous success based on the miniaturization and the high information content that this format provides. The DNA microarray is now a standard technology and customized arrays with 10^4^–10^6^ discrete sequences are readily available. Screening for glycan binding in a microarray format has also proven extremely valuable in glycobiology [46–47]. Based on earlier reports that small molecules [48–49] and protein microarrays [50] can be obtained by hybridization of PNA-tagged libraries, Chevolot and co-workers first reported the use of glycan–DNA conjugates to display glycans in a spatially addressable array format [27]. A fluorophore (Cy3) was used to quantify the immobilized conjugate on the array. In a pilot experiment, a galactose binding lectin (RCA_120_) was applied to confirm the spatial resolution upon hybridization (Figure 6).

**Figure 7:**
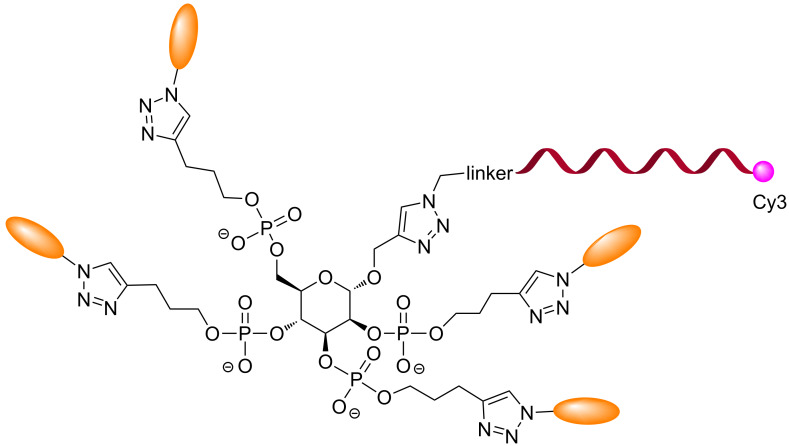
Multivalent sugar-core glycoconjugate DNA.

The same group extended this concept with conjugates built on a glucose scaffold displaying up to four units of the glycans (Figure 7) to generate homo- or heteroglycan cluster. These conjugates were used for hybridization to DNA arrays and screened against lectins from pathogenic *P. aeruginosa* (PA2L and LecA) [51–53].

**Figure 8:**
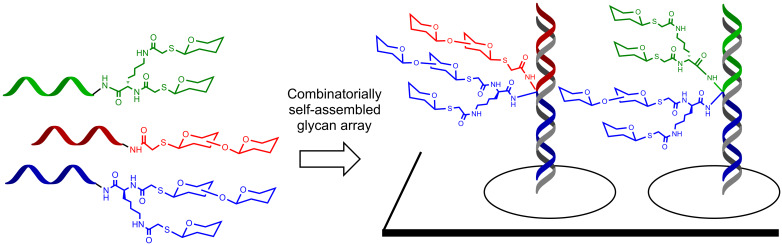
Combinatorial self-assembly of PNA glycoconjugates on a DNA microarray.

Our group used DNA microarrays to combinatorial pair diverse PNA-tagged glycan conjugates displayed at adjacent hybridization sites to produce assemblies emulating the diversity of di-, tri- and tetra-antennary glycans (Figure 8) [39]. Using two sets of 25 PNA conjugates, an array of 625 unique assemblies was produced. Importantly, screening different lectins (ConA or peanut lectin) indicate a synergy between the paired fragments with a composition consistent with the known selectivity of the lectins.

**Figure 9:**
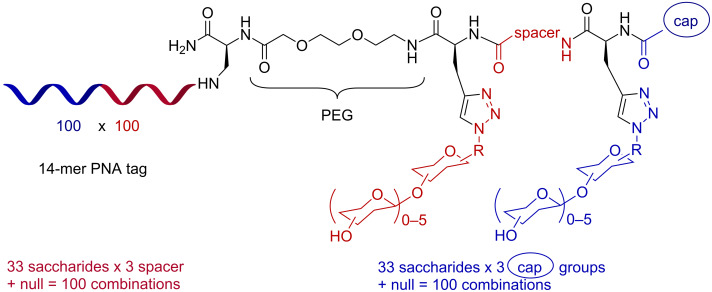
General scheme of the 10,000 member PNA-encoded glycoconjugate library.

**Figure 10:**
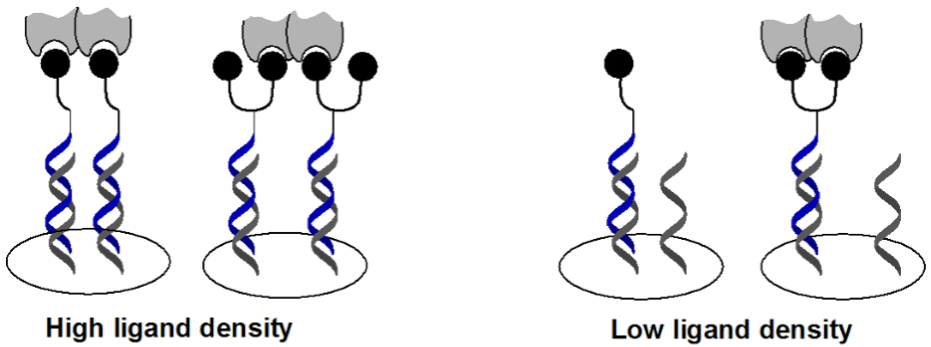
Oligomeric interaction with arrayed mono- and divalent ligands (represented as the black spheres) as a function of surface density.

More recently, we reported a combinatorial synthesis of a more diverse library of PNA-encoded glycoconjugates (10,000 members) [41]. The combinatorial synthesis was performed using two sequential CuAAC conjugations with 33 diverse glycosyl azides separated by 3 different peptide spacers and capped by 3 different aryl groups (Figure 9). The fact that the library can be prepared by mix and split synthesis and reformatted in a spatially addressable microarray by simple hybridization [48] greatly facilitates access to diverse glycoconjugate arrays. Screening the library against a panel of seven different lectins (ConA, Bc2LA, BambL, BSL, LecA , StxB and MAL) showed a distinct binding selectivity in each case for a conjugate of two glycans (relative to control with a single glycan) with a unique linker and capping group combination thus establishing the synergy of interaction between the glycan units and the distinct spatial arrangement conferred by the different linkers. This library represents the largest array of heteroglycan conjugates reported to date. Based on the results obtained with the screen for LecA, a lectin intimately involved in the pathogenicity of *P. aeruginosa*, a focused library displaying two galactose mono- or disaccharides with different linkers was synthesized in order to optimize affinity of a conjugate interacting with one face of the lectin [42]. LecA is a tetrameric protein with two binding sites on each face of the oligomer. An important question in microarray-based affinity screens with proteins involved in oligomeric interactions is whether a high-intensity interaction observed on the array results from a unique high-affinity ligand or from multiple lower affinity ligands due to the high surface density of these in the microarray format (Figure 10). Comparing the binding of a divalent ligand with its monomeric counterpart at decreasing ligand concentration showed a faster decay of binding for the monomeric ligand consistent with the speculation that, at high ligand concentration, the density was sufficiently high for the lectin to interact with multiple ligands across different hybridization sites. Notably, this work led to the discovery of a high-affinity ligand (*K*_D_ = 82 nM) that was effective at inhibiting bacterial penetration in epithelial cells.

## Conclusion

It is now well-established that nucleic acid-based assemblies can be used to display glycans with a synergy between the interactions of the individual ligands binding with a target. Progress in this area has paralleled similar developments with small molecule conjugates [48,54–63]. Technological developments in conjugation chemistry and solid-phase synthesis have enabled the introduction of complex glycans with modest synthetic investments to obtain the suitably functionalized glycans. Furthermore, methods to access large libraries of peptidoglycan conjugates with nucleic acid tags have been reported opening new horizons in the diversity space that can be screened for this important compound class. The fact that assemblies can be prepared with control over the ligand spacing, combination and valence is empowering. While the geometry of architectures tested has remained fairly simple thus far (linear or 3-way junction), progress in DNA-based nanoassemblies will likely fuel further advances in the area of hybridization-based glycan displays. It can also be anticipated that these assemblies will be used in increasingly more complex systems extending beyond simple affinity measurements, paving the way towards diagnostic or therapeutic applications. We hope that the examples presented in this review will encourage researchers in glycoscience to embrace and further develop the use of glycan display by programmed assemblies.
